# Characterization of retinal microvasculature in acute non-arteritic anterior ischemic optic neuropathy using the retinal functional imager: a prospective case series

**DOI:** 10.1186/s40662-018-0126-x

**Published:** 2019-01-06

**Authors:** Amanda D. Henderson, Hong Jiang, Jianhua Wang

**Affiliations:** 1 Wilmer Eye Institute, Johns Hopkins University School of Medicine, 600 N. Wolfe Street, Wilmer 233, Baltimore, MD 21231 USA; 2 Bascom Palmer Eye Institute, University of Miami, Miller School of Medicine, 900 NW 17th Street, Miami, FL 33136 USA

**Keywords:** Retinal functional imager, Non-arteritic anterior ischemic optic neuropathy, Blood flow velocity

## Abstract

**Background:**

Non-arteritic anterior ischemic optic neuropathy (NAION) is the most common cause of acute optic neuropathy in patients over 50 years of age, and many affected individuals are left with permanent visual deficits. Despite the frequency of NAION and its often devastating effects on vision, no effective treatment has been established. Further understanding of the acute vascular effects in NAION, using advanced ophthalmic imaging techniques like the retinal function imager, may shed light on potential treatment targets.

**Methods:**

Five patients with acute NAION underwent retinal functional imaging within 2 weeks of the onset of their visual symptoms, and at 1 month and 3 months after onset. Average arteriolar and venular blood flow velocities were calculated for each eye at each time point. The Wilcoxon rank sum test was used to compare blood flow velocity results with a normative database.

**Results:**

The average arteriolar blood flow velocity in the normative group was 3.8 mm/s, and the average venular blood flow velocity was 3.0 mm/s, versus 4.1 mm/s and 2.7 mm/s, respectively, in the NAION-affected group at presentation. Average arteriolar blood flow increased slightly to 4.2 mm/s one month after the acute NAION event, then decreased to 3.8 mm/s three months after the event. Average venular blood flow velocity was 2.8 mm/s 1 month after the NAION event and 2.7 mm/s 3 months after the event. Differences in blood flow velocity between the NAION-affected and control groups were not statistically significant at any time point; however, there was a trend toward increasing blood flow velocity initially after an NAION, with a decrease over time.

**Conclusions:**

This study demonstrates the feasibility of retinal function imaging to quantify macular blood flow velocity in patients with acute NAION. There were no statistically significant differences in blood flow velocity detected between NAION-affected eyes and healthy controls at any of the time points examined; however, there was a trend toward an increase in both arteriolar and venular BFV subacutely, then a decrease in the chronic phase after NAION, which could be suggestive of a mechanism of attempted compensation in the setting of acute ischemia.

## Background

**Fig. 1:**
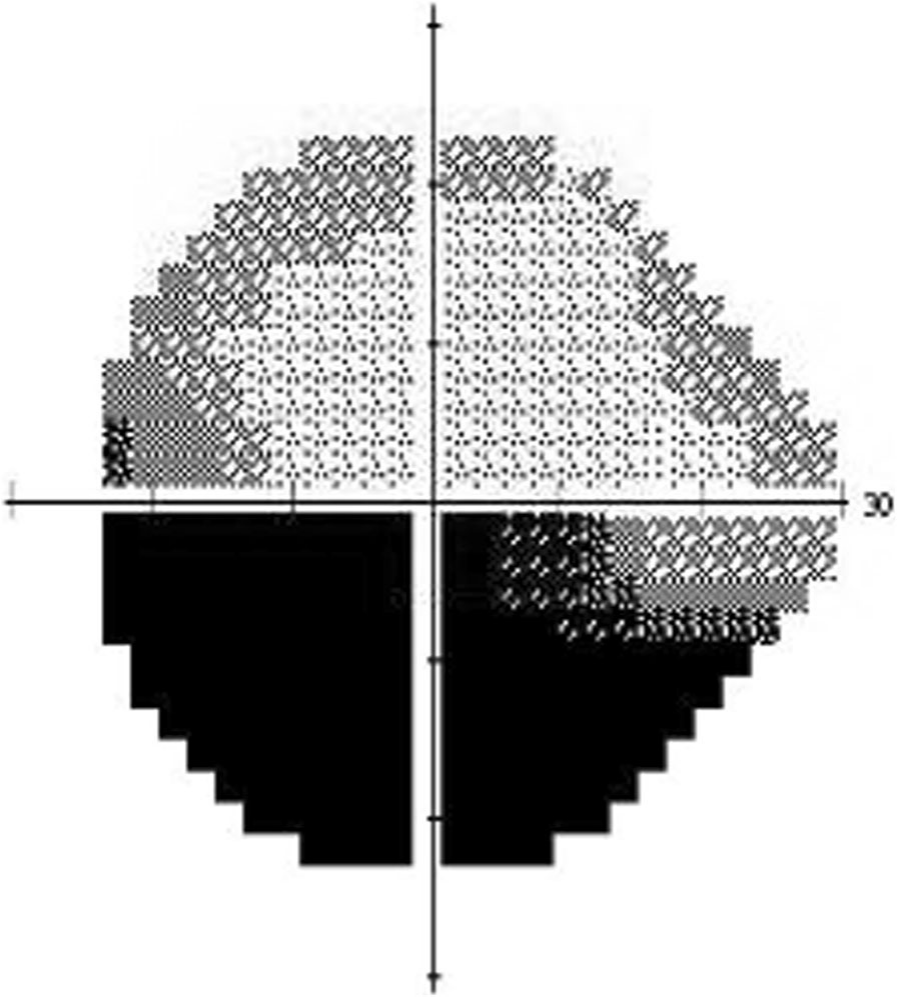
Inferior altitudinal visual field defect

Non-arteritic anterior ischemic optic neuropathy (NAION) is the most common cause of acute optic neuropathy in patients over age 50 years, and many affected individuals suffer from permanent visual deficits. [1] The primary mechanism for NAION is thought to be acute hypoperfusion of the optic nerve from the posterior ciliary artery branches. [2] Systemic risk factors for the development of NAION include diabetes, systemic hypertension, nocturnal hypotension, blood loss, obstructive sleep apnea, and hypercoagulable disorders. [3–9] Presenting features include painless monocular visual loss, a relative afferent pupillary defect in the involved eye, and optic disc edema, often with peripapillary hemorrhages. Visual field testing commonly shows altitudinal defects, although other patterns of visual field loss can occur (Fig. 1). After resolution of edema, disc pallor, often in a segmental pattern, typically results. Although steroid treatment in the acute phase has been recommend by some experts, [2] no treatment has proved effective for this condition.

Further understanding of the acute vascular effects of NAION may shed light on potential treatment targets. Flow impairment in the prelaminar optic disc in the setting of an acute NAION has been demonstrated. [10] Color Doppler has shown decreased flow velocity in the central retinal artery in acute NAION; however, this technique is limited by its inability to account for vessel diameter. [11] We hypothesized that, similar to posterior ciliary arteries, branches from the central retinal artery also would have flow alterations in the setting of NAION. Effects on blood flow in these arterioles can be measured with the retinal functional imager (RFI). The RFI allows for calculation of flow velocities in retinal arterioles and venules, accounting for the width of individual vessel segments, which provides information regarding the perfusion provided by these retinal vessels. To our knowledge, this report is the first describing the effects of NAION on macular blood flow velocity (BFV), as characterized by RFI scanning.

## Methods

This study was approved by the Institutional Review Board of the University of Miami and adhered to the tenets of the Declaration of Helsinki for research on human subjects. Informed consent was obtained from all participants. The aim of this study was to characterize the retinal microvasculature in patients with acute NAION using advanced imaging with the RFI.

Five patients were recruited from the outpatient neuro-ophthalmology clinic at the Bascom Palmer Eye Institute over a 6-month time period from October 2015 to April 2016. Inclusion criteria included a diagnosis of NAION by a Bascom Palmer neuro-ophthalmologist, with onset of visual symptoms in the involved eye within 2 weeks prior to presentation, and ability and willingness to provide informed consent in English. Patients were excluded if significant media opacity, such as cataract, prevented adequate imaging with the RFI.

Participants underwent routine clinical testing at their initial appointments, including visual acuity testing, fundus photographs, optical coherence tomography (OCT) of the ganglion cell layer-inner plexiform layer and retinal nerve fiber layer using the Zeiss Cirrus OCT (Carl Zeiss Meditech, Inc.), and Humphrey visual fields (30–2 Swedish Interactive Thresholding Algorithm standard program). All testing was performed in both the affected and unaffected eyes. Additionally, on the initial imaging day, imaging was performed with the RFI. Each subject in this study was treated at the discretion of her/his neuro-ophthalmologist and not according to a treatment protocol. Some patients were offered steroid treatment, after discussion regarding lack of evidence for benefit, if they had no comorbidities (e.g., poorly-controlled diabetes mellitus) that precluded steroid use. Any treatment was documented at the initial appointment and throughout the follow up period. Similar testing, including RFI, was performed initially within 2 weeks of symptom onset, then again at 1 month and 3 months after the onset of vision loss. Two patients withdrew after the 1-month imaging session, and 3 patients completed the planned imaging sessions.

**Fig. 2:**
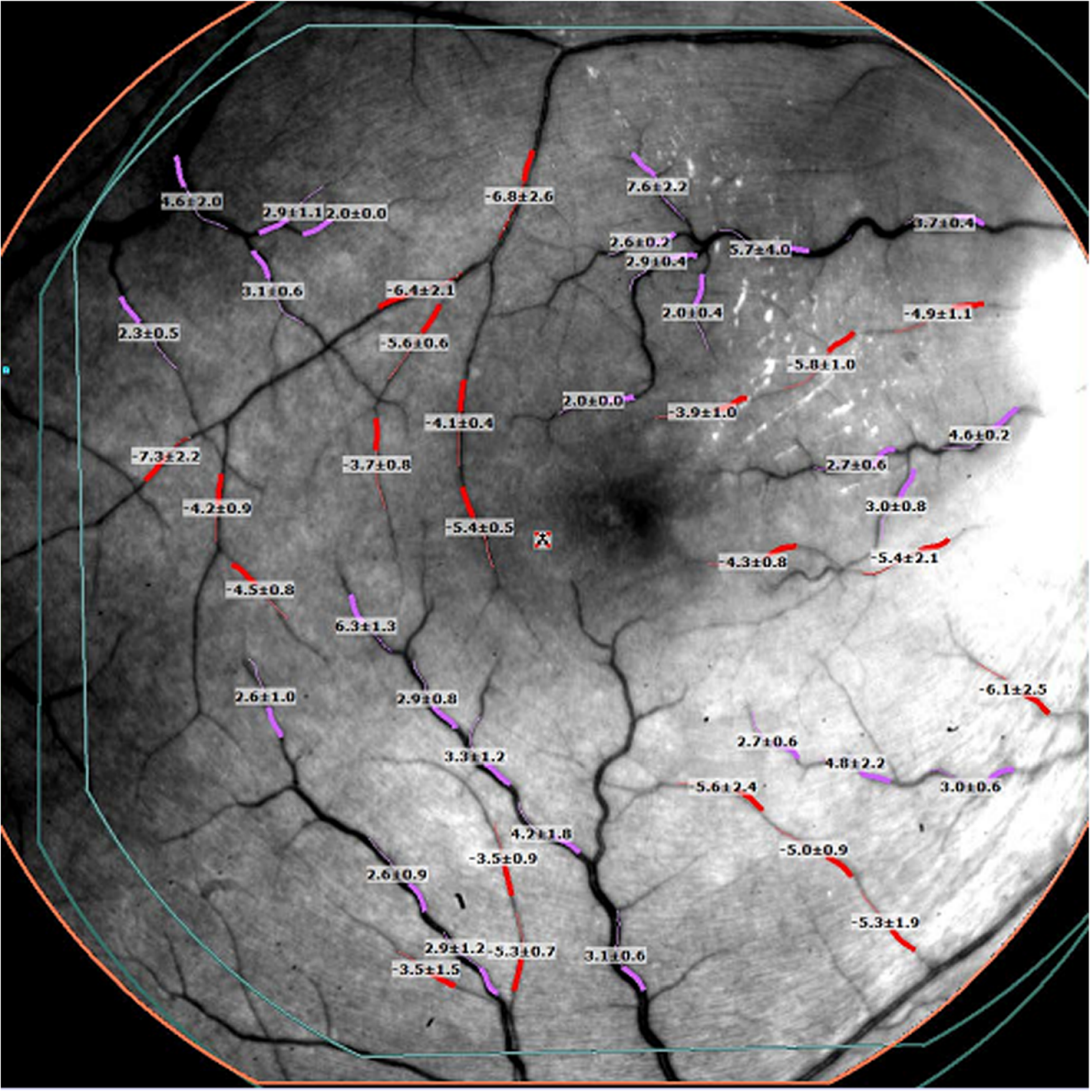
Blood flow velocity map as produced by the retinal functional imager. Red demonstrates arteriolar blood flow toward the macula, and purple demonstrates venular blood flow away from the macula

RFI is an advanced ophthalmic imaging technique, which measures retinal microvascular function, specifically BFV. A large field of view up to 35 degrees (imaged area of 7.3 mm^2^), centered on the fovea, can be obtained by the RFI. RFI previously has been described in detail [12–15]. The images in this study were acquired using a stroboscopic light source and a high-resolution digital camera (Topcon Medical Systems, Inc., Oakland, NJ, USA). Pupillary dilation was required prior to image acquisition. A rapid series of retinal images was captured using red-free illumination and an interference filter with transmission centered at 548 nm and a bandwidth of 75 nm. To control for the effect of cardiac pulsation on BFV, a probe was attached to each subject’s finger, allowing image acquisition to be synchronized to a selected phase of the pulse pattern. After the digital pictures were captured, they were stored and then processed using differential imaging, which directly detects moving erythrocytes in retinal vessels as small as 4 μm in diameter. The measured velocities in the secondary and tertiary branches of the arterioles and venules were also recorded such that a BFV map was depicted for the arterioles (flowing toward the fovea) and venules (flowing away from the fovea) (Fig. 2).

Descriptive statistics were calculated using Microsoft Excel and XLSTAT (Addinsoft, New York, NY). The Wilcoxon rank sum test was used to compare results with a normative database, and statistical significance was set at *p* < 0.05. The data forming the normative database were collected from healthy control subjects, none of whom had a history of diabetes, hypertension, or obstructive sleep apnea.

## Results

Enrolled subjects included 3 females and 2 males, ranging in age from 46 to 70 years, with a mean age of 59 years. Underlying risk factors for NAION included hypertension in 4 participants, obstructive sleep apnea in 2 participants (1 of whom was not using a continuous positive air pressure device), evening use of blood pressure-lowering medications in 2 participants, and diabetes mellitus in 3 participants. One participant had no known underlying risk factors for NAION. One participant reported headaches, and the remaining 4 participants denied symptoms concerning for giant cell arteritis, including headache, jaw claudication, scalp tenderness, weight loss, fever, and polymyalgia rheumatica. Erythrocyte sedimentation rate and C-reactive protein were normal in 4 subjects and were not checked in the 46-year-old due to his young age. Magnetic resonance imaging of the brain and orbits was normal in 3 participants and was not performed in the remaining 2. At presentation, Snellen visual acuity in the affected eye ranged from 20/25 to 20/350. Three subjects received treatment with prednisone, while the other 2 did not receive any treatment. Individual findings are shown in Table 1.

**Table 1:** Baseline Characteristics of Study Subjects

Subject	Age (years)	NAION Risk Factors	Initial Snellen Acuity	GCA symptoms	Labs	MRI	Treatment
1	64	None	20/30	Headache	ESR/CRP/plts normal	Negative	Prednisone
2	70	HTN, OSA, BP meds in evening	20/350	None	ESR/CRP/plts normal	Negative	Prednisone
3	46	DM, HTN, OSA not using CPAP, BP meds in evening	20/40	None	None	Negative	None
4	54	DM, HTN	20/25	None	ESR/CRP/plts normal	Not performed	None
5	63	DM, HTN	20/70	None	ESR/CRP wnl	Not performed	Prednisone

Abbreviations: *BP* = blood pressure; *CRP* = c-reactive protein; *ESR* = erythrocyte sedimentation rate; *GCA* = giant cell arteritis; *HTN* = hypertension; *MRI* = magnetic resonance imaging; *NAION* = non-arteritic anterior ischemic optic neuropathy; *OSA* = obstructive sleep apnea; *plts* = platelets; *wnl* = within normal limits

**Fig. 3:**
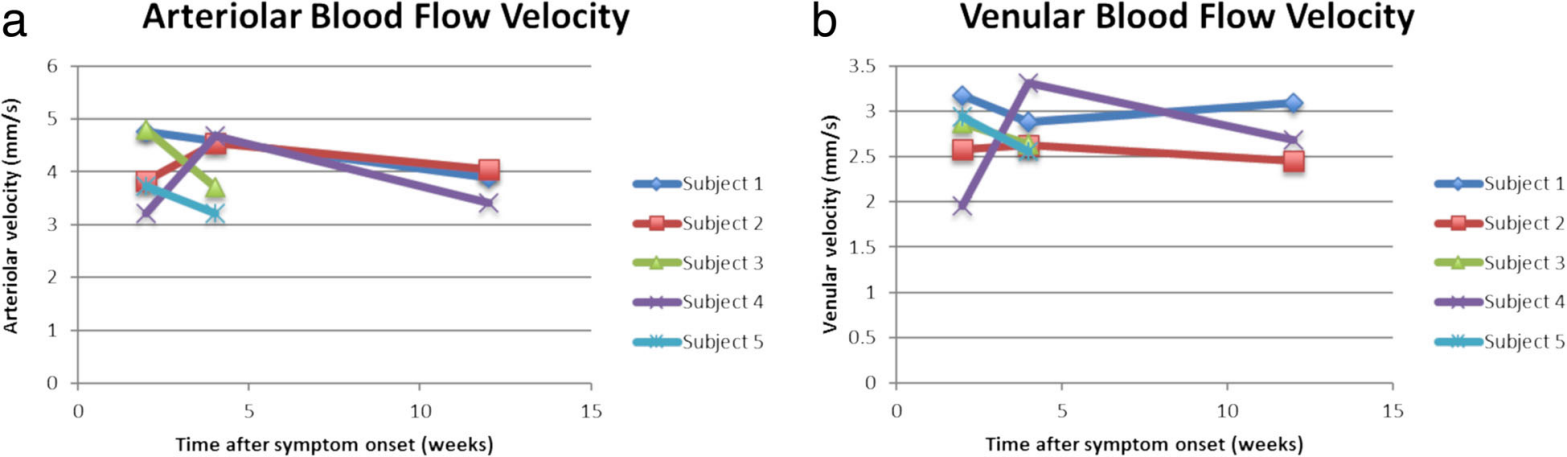
Arteriolar (**a**) and venular (**b**) blood flow velocities for individual subjects, as measured by the retinal functional imager

**Fig. 4:**
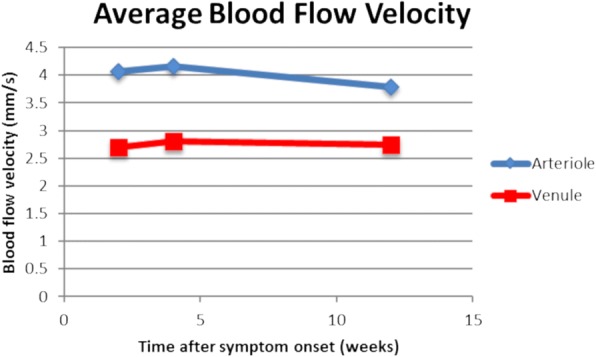
Average arteriolar and venular blood flow velocities (BFV). Notice the trend of increasing BFV initially after non-arteritic anterior ischemic optic neuropathy, with a decrease in BFV over time thereafter

BFVs attained from RFI in participants with NAION were compared with those in a normative database of 58 healthy controls, including 25 males and 33 females, ages 18–70 (mean 35). The average arteriolar BFV in the normative database group was 3.8 mm/s (SD 0.80), and the average venular BFV was 3.0 mm/s (SD 0.57), versus 4.1 mm/s (SD 0.70) and 2.7 mm/s (SD 0.47), respectively, in the NAION-affected group at presentation. Average arteriolar BFV increased slightly to 4.2 mm/s (SD 0.65) 1 month after the acute NAION event, then decreased to 3.8 mm/s (SD 0.33) 3 months after the event. Average venular BFV was 2.8 mm/s (SD 0.31) 1 month after the NAION event and 2.7 mm/s (SD 0.32) 3 months after the event. Differences in BFV between the NAION-affected and control groups were not statistically significant at any time point. There was, however, a trend of increasing BFV initially after an NAION, with a decrease in BFV over time. Average arteriolar BFV at the presentation, 1-month, and 3-month time points was 4.1 mm/s (SD 0.58), 4.1 mm/s (SD 0.78), and 4.0 mm/s (SD 0.11), respectively, in the prednisone-treated patients, versus 4.0 mm/s (SD 1.1), 4.2 mm/s (SD 0.69), and 3.4 mm/s, respectively, in the non-prednisone treated patients. Average venular BFV at the time of presentation, 1-month, and 3-month time points was 2.9 mm/s (SD 0.29), 2.7 mm/s (SD 0.17), and 2.8 mm/s (SD 0.45), respectively, in prednisone-treated patients, versus 2.4 mm/s (SD 0.64), 3.0 mm/s (SD 0.47), and 2.7 mm/s, respectively, in the non-prednisone treated patients. While these data suggest slightly more decrease in BFV in the non-prednisone-treated subjects at the 3-month time point, the small number of study participants limits conclusions regarding the effect of steroid treatment on BFV. Arteriolar and venular BFVs for the individual study subjects are shown in Fig. 3. Average BFVs are shows in Fig. 4.

## Discussion

RFI has demonstrated decreased retinal BFV in diseases primarily affecting the macula, including central serous chorioretinopathy, non-proliferative diabetic retinopathy, and age-related macular degeneration, as well as in more diffuse retinopathies including retinitis pigmentosa. [16–19] Although the application of RFI to optic neuropathies has been limited, evaluation has been performed in patients with primary open angle glaucoma (with visual field defects) and in patients with pre-perimetric glaucoma (in the absence of visual field defects). Only pre-perimetric glaucoma patients were found to have significant alterations in BFV, with a higher BFV than controls, suggesting a possible attempt at compensatory vascular alterations early in the disease course, which no longer were present in more advanced disease with visual field loss. [20] It is possible that our data showing a trend toward an initial increase in BFV 1 month after an acute NAION, followed by a decrease in BFV by 3 months after the event, may be explained by a similar mechanism of attempted compensation in the setting of acute ischemia, which then resolves over time as irreparable structural damage occurs.

The primary pathophysiology of NAION is thought to involve the short posterior ciliary arteries supplying the optic nerve; however, reactive mechanisms after the initial insult may potentiate retinal ganglion cell damage. Therefore, it is reasonable to consider that alterations in the retinal vascular bed that supplies the inner retina, including the retinal ganglion cell layer, may also play a role. OCT angiography has been used to characterize optic disc perfusion in chronic NAION, and results have shown a reduction in disc perfusion in eyes that had been affected by NAION, compared with healthy controls. [21–24] However, due to swelling of the peripapillary retinal nerve fiber layer in the acute and subacute phases of NAION, peripapillary OCT angiography scan quality is reduced, limiting the interpretation of imaging of the peripapillary region. [23, 24] Therefore, the vessels of the central macula are more accessible for evaluation of blood flow in this setting.

Limitations of our study include the small sample size and lack of a matched control group for comparison. Although NAION is the most common acute optic neuropathy in patients over age 50, it remains relatively rare, with an incidence of 2.3 to 10.2 cases per 100,000 people age 50 and older. [1, 2, 10, 25, 26] Additionally, delayed presentation to a neuro-ophthalmologist reduces the number of patients affected by NAION evaluated in the acute or subacute phase. Therefore, attaining a large cohort of patients acutely affected by NAION at a single center is difficult.

Another potential limitation is that the average age of the patients in our normative database was younger than the average age of study patients, which could confound the findings. Of note, a negative correlation between age and venular BFV was demonstrated after age 40 years; however, no such correlation was demonstrated for arteriolar BFV, suggesting that age matching may not be required to compare arteriolar BFVs. [27] Direct age matching from the normative sample was not possible due to inadequate data samples. The initial plan to address this shortcoming was to use the subjects’ own fellow eyes as the control group; however, 3 of the 5 patients had a significant ocular abnormality in the fellow eye (1 with remote NAION; 1 with subacute bilateral, sequential NAION; and 1 with a large macular scar), which prohibited this comparative approach. Expansion of the normative database with further collection of RFI data among older individuals may help to address this problem in the future.

Finally, there is a lack of conclusive evidence regarding the efficacy of steroids to treat an acute NAION. However, as subjects included in this study were treated at the discretion of their individual neuro-ophthalmologists and not as part of a pre-determined treatment protocol, some subjects were offered steroid treatment; 3 of the 5 subjects received prednisone treatment. It is possible that prednisone use may have affected the reactive mechanisms following the ischemic event in NAION, and it is possible that retinal blood flow could have been affected.

## Conclusions

This study demonstrates the feasibility of using the RFI to quantify BFV in the macula of patients with acute NAION with optic disc swelling. The sample size was limited, but there were no statistically significant differences in BFV detected between NAION-affected eyes and healthy controls at any of the time points examined. There was a trend toward an increase in both arteriolar and venular BFV subacutely, then a decrease in the chronic phase after NAION. Further studies with larger sample sizes and case control matching will be required to confirm this finding.

## Abbreviations

BFV: Blood flow velocity
NAION: Non-arteritic anterior ischemic optic neuropathy
OCT: Optical coherence tomography
RFI: Retinal functional imager
